# Low dose compared to variable dose Warfarin and to Fondaparinux as prophylaxis for thromboembolism after elective hip or knee replacement surgery; a randomized, prospective study

**DOI:** 10.1186/s12959-015-0062-0

**Published:** 2015-10-07

**Authors:** Murray M. Bern, Diane Hazel, Elizabeth Deeran, John R. Richmond, Daniel M. Ward, Damon J. Spitz, David A. Mattingly, James V. Bono, Ronna H. Berezin, Laura Hou, Gerald B. Miley, Benjamin E. Bierbaum

**Affiliations:** Departments of Medicine, New England Baptist Hospital, Boston, MA USA; Orthopedic Surgery, New England Baptist Hospital, Boston, MA USA; Diagnostic Radiology, New England Baptist Hospital, Boston, MA USA; Research, New England Baptist Hospital, Boston, MA USA; Harvard Medical School, Boston, MA USA; University of New Mexico Cancer Center, 1201 Camino de Salud, Albuquerque, NM 87131 USA; Tufts University School of Medicine, Boston, MA USA

**Keywords:** Arthroplasty, Fondaparinux, Hip replacement, Knee replacement, Prophylaxis, Thrombophlebitis, Warfarin, Deep vein thrombosis, Pulmonary embolus

## Abstract

**Background:**

Deep vein thrombosis (DVT) and pulmonary emboli (PE), known together as venous thromboembolic (VTE) disease remain major complications following elective hip and knee surgery. This study compares three chemoprophylactic regimens for VTE following elective primary unilateral hip or knee replacement, one of which was designed to minimize risk of post-operative bleeding.

**Methods:**

Patients were randomized and stratified for hip vs. knee to receive A: variable dose warfarin (first dose on the night preceding surgery with subsequent target INR 2.0–2.5), B: 2.5 mg fondaparinux daily starting 6–18 h postoperatively, or C: fixed 1.0 mg dose warfarin daily starting 7 days preoperatively. All treatments continued until bilateral leg venous ultrasound day 28 ± 2 or earlier upon a VTE event. The study examined primary endpoints including leg DVT, PE or death due to VTE and secondary endpoints including effects on D-dimer, estimated blood loss (EBL) at surgery and hemorrhagic complications.

**Results:**

Three hundred fifty-five patients were randomized. None was lost to follow-up. Taking 1.0 mg warfarin for seven days preoperatively did not prolong the prothrombin time (PT). Two patients in Arm C had asymptomatic distal DVT. One major bleed occurred in Arm B and one in Arm C (ischemic colitis). Elevated d-dimer did not predict delayed VTE for one year.

**Conclusions:**

Fixed low dose warfarin started preoperatively is equivalent to two other standards of care under study (95 % CI: -0.0428, 0.0067 for both) as VTE prophylaxis for the patients having elective major joint replacement surgery.

**Trial registration:**

ClinicalTrials.gov identifier # NCT00767559

FDA IND: 103,716

## Background

When initiated prior to elective surgery low dose warfarin has been demonstrated prophylactic capacity against VTE with low risk of hemorrhage [1–14]. Warfarin when given at 1 mg per day is known to increase serum under-gamma-carboxylated osteocalcin and plasma under-gamma-carboxylated prothrombin, without change of prothrombin times or factor VII activity [15]. It is critical in this pharmacologic format that low dose warfarin be initiated prior to the surgical trauma, thus allowing time for warfarin to partially suppress the carboxylation of glutamic acid residues attached to the cores of factors II, VII, IX and X [16]. This sequentially reduces their calcium ion dependent function [16–21] It is this mechanism which we believe reduces the efficiency of calcium binding, thus slowing the efficiency of clotting but not so much as to cause full anticoagulation.

This study is a continuum of studies predicated upon the concept that the clotting system can be kept in a homeostatic balance, making it possible to avoid added risk of bleeding while protecting patients from thromboembolism [1–14]. Among these studies was a prospective pilot study of 100 patients in which patients planning total hip replacement were randomized between two arms, one with warfarin starting with 5.0 mg the night prior to surgery followed by variable dose warfarin (target PT 1.3–1.5 x normal) and the other using 1.0 mg of warfarin beginning seven days prior to surgery, and both continuing for 30–45 days. There was no difference for incidence of venous thrombosis [8]. Another was a retrospective study of patients undergoing primary (*n* = 833) or revision (*n* = 170) hip replacement arthroplasty who received 1.0 mg of warfarin for seven days prior to surgery, variable dose warfarin while in hospital (target INR 1.5–2.0) followed by1.0 mg daily until follow-up at 30–45 days. Of 1,003 patients, only 3 (0.3 %) had symptomatic VTE [11].

This current investigator-initiated, single-institution, prospective, randomized, open-label assessor-blind study study compared fixed low dose of warfarin to two control arms as chemoprophylaxis against VTE among patients undergoing elective primary hip or knee replacement. Fixed low dose warfarin was initiated 7 days prior to surgery so as to allow the necessary time for its effects to be in place by the time of surgery. All patients received the same optimal non-pharmacologic support. We hypothesized that the three chemotherapeutic regimens would yield equivalent clinical findings for these patients. If confirmed as effective the fixed low dose regimen should have minimal hemorrhagic potential and would require less laboratory support, but with less cost than the modern novel oral anticoagulants that are also available [22].

## Methods

### Research design

This was a prospective, randomized three-arm study including two control arms (warfarin and fondaparinux in standard doses) and a third arm using warfarin at 1.0 mg per day starting seven days before surgery. Therapy was continued until a VTE event occurred or until 28 ± 2 days from day of surgery when bilateral leg ultrasounds were performed. (see Table 1) All patients were called 6 and 12 months following their surgery to detect any delayed complications or VTE events.

**Table 1:** Research design

ARM A:	
Variable dose warfarin:	
	5.0 mg beginning the night before surgery, followed by 5.0 mg the PM of surgery, and then variable daily dose, (target INR 2.0-2.5) until day 28 ± 2 follow-up.
ARM B:	
Fondaparinux:	
	2.5 mg daily starting 6 or more hours following surgery, but no later than 6 AM the next day, or 6-8 h after epidural catheter removal; continued until day 28 ± 2
ARM C:	
Fixed low dose warfarin	
	1.0 mg daily beginning 7 days preoperatively, and continued at 1.0 mg daily until day 28 ± 2 follow-up.

Bilateral, full leg, venous Doppler and ultrasound analysis 28 ± 2 following surgery

Follow-up phone calls at 6 and 12 months following surgery

The primary endpoint was composite DVT, PE or death due to VTE. Secondary endpoints included frequency of proximal vs. distal DVT, estimated blood loss (EBL) at surgery, and hemorrhagic complications.

The hospital’s Institutional Review Board (IRB) approved this study. All patients gave signed informed consent.

### Randomization

Total randomized patient allocations were to be at 1:1:1 ratio for each arm at end of study. The randomization was stratified for hip vs. knee surgery. A member of the pharmacy department pulled randomized cards as prepared by the statisticians. The results were communicated to the Clinical Research Coordinator (CRC), who then shared the results with the patients. Notice of consent was place in the patient’s chart, along with pre-and postoperative orders. Study diaries were then given to all consenting patients.

### Patient recruitment

Participants were recruited from among patients over 20 years of age planning elective primary unilateral total hip or knee replacement surgery at an orthopedic specialty hospital. Exclusion criteria are demonstrated in Table 2. There were no gender, ethnicity or race-based restrictions.

**Table 2:** Exclusion criteria

1. Abnormal platelet count, prothrombin time (PT) or partial thromboplastin time (PTT)
2. Surgery for acute fracture (<4 weeks), septic joint, or extraction arthroplasty
3. History of VTE or documented hypercoagulation syndrome
4. Increased risk of hemorrhage, as from active gastric ulcer or urinary tract bleed within the last year
5. Hemorrhagic stroke; brain, spinal, or ophthalmologic surgery in previous 6 months
6. Liver enzymes or bilirubin greater than 2 x normal
7. Decreased renal function with GFR <30 ml/min.
8. Cancer in last year, other than localized cancers of the skin
9. Requires chronic anticoagulation
10. Requires chronic platelet function suppressive therapy
11. Prior adverse reaction to any of the study drugs
12. Uncontrolled hypertension
13. BMI >42
14. Pregnancy

### Surgical and postoperative care

Eleven orthopedic surgeons participated in this study. The surgical, anesthesia and postoperative procedures were according to the standardized procedures used in our hospital. Patients had early post-operative ambulation. All patients wore pneumatic compression stockings while in-patient. Elastic compression stockings were prescribed to be used after discharge until the follow-up ultrasounds. Hydroxyethyl starch (HES) 6 % was allowed intraoperatively for case specific reasons. Use of platelet function suppressive drugs, such a nonsteriodal anti-inflamatory drugs (NSAIDs), was discouraged but not prohibitied by the protool.

### Laboratory monitoring

All patients receiving warfarin in hospital had PT measured daily. Thereafter, patients discharged on variable dose warfarin had PT measurements twice per week. Warfarin doses were prescribed by the hospital’s Coumadin Hotline personnel with target International Normalized Ratio (INR) 2.0–2.5. The patients on the 1 mg warfarin study arm had PT measured weekly. D-dimer was tested by flocculation and by quantitative assays upon admission and at the 28 ± 2 day follow-up visits with the results are reported as negative (0–500 μg/mL), indeterminate (500–1,000 μg/mL), or positive (>1,000 μg/mL). (Diagnostica Stago, American Bioproducts Co., Parsippany, New Jersey 07054).

### Patient monitoring

Clinical Research Coordinators (CRCs) visited patients daily during inpatient hospitalization. After discharge the CRC’s called the patients weekly to monitor compliance and for any complications. The patients were met by the CRC’s again at the time of the follow-up ultrasound. Additionally, patients kept daily diaries of the doses and times of administration of their study drug along with any other drugs they were taking, and any complications and return visits to doctors or hospitals. Telephone interviews were conducted by the CRCs after 6 and 12 months to detect any delayed complications or VTE events.

### Examination for deep vein thrombosis and pulmonary embolus

Patients underwent bilateral duplex sonography to evaluate any clinical suggestions of DVT or if asymptomatic on day 28 ± 2 following surgery. Gray scale, color, and spectral Doppler sonography with augmentation were performed of the bilateral common femoral veins, deep femoral veins, superficial femoral veins, popliteal veins, proximal greater saphenous veins, anterior and posterior tibial veins, and peroneal veins. Three highly trained technologists were involved in this study applied the Practice Guidelines and Technical Standards of the American College of Radiology, revised 2010. Ventilation/perfusion lung scan or computerized axial tomography angiogram was performed for evaluation of symptoms of pulmonary embolus. Radiology technicians and the radiologists were blinded to patient randomization.

### Quality assurance

The Protocol Committee reviewed data weekly. The Data and Safety Monitoring Board (DSMB) examined blinded data every three months with authority to stop the study if deemed necessary.

### Participant retention

Patients were removed from the study drug, but not from the study, if they reached a composite endpoint or if they elected to discontinue the study drug, including those patients who required change in their anticoagulation for ancillary medical reasons.

### Protocol violations and adverse events

The Protocol Committee reviewed in blinded fashion all potential protocol violations and adverse events. The likelihood that there be a relationship between the reported events and the study drugs was assigned as being not related, unlikely related, possibly related, probably related or related. A major adverse event was defined as one that required medical or surgical intervention while in hospital, delayed discharge, or prompted return to office or hospital for reevaluation (whether or not intervention was required).

Hemorrhagic complications were classified as major or minor. A minor hemorrhage was one that was observed but required no intervention, such as epistaxis or venipuncture site hematoma. It may have been significant enough to warrant discharge delay or prompt a visit to the doctor or hospital, but with no intervention required. A major hemorrhage would include any fatal bleed or symptomatic bleed into critical area or organ requiring intervention.

Any major adverse event was reported to the IRB, DSMB and if required to the Federal Drug Administration.

### Statistical methods

The study analysis was based upon intention to treat and thus all consented patients were included in the analysis. The primary analysis was based on a dichotomous outcome (i.e. VTE–yes/no) with VTE defined as the composite of DVT or PE or death. Estimates of how many patients were required in each arm to detect a difference were based on prior reported experiences in similar studies. With the goal to demonstrate equality for Arm C (upper one-sided 95 % confidence limit for treatment Arm C minus standard treatment arms A and B with the delta not to exceed 15 %), conservative estimates of DVT rates from prior studies and powered to 90 %, it was concluded that 110 patients were needed per group. As there were fewer endpoint events than expected in the three arms, the Odds Ratio was calculated for the composite endpoints using an exact logistic regression conditional analysis, a technique to be used when the outcomes variable have no observations. The non-inferiority analysis was completed using the Wald non-inferiority test.

A statistician analyzed the outcomes for differences between the study arms, differences within subsets (hip and knee arthroplasty), and differences within arms A, B and C for the primary and secondary endpoints. Multivariate analysis is reported for age, gender, body mass index (BMI), and use of hydroxyethyl starch or NSAIDs. Primary outcome data are stratified for hip vs. knee surgery, symptomatic vs. asymptomatic, and popliteal vein and above (proximal) vs. more distal veins. Secondary endpoints were analyzed for the patients as a total cohort. The *p*-values for all comparative results were calculated.

This was an open label, assessor-blinded study. All authors had access to the data.

## Results

### Patient recruitment, randomization and retention

**Fig. 1:**
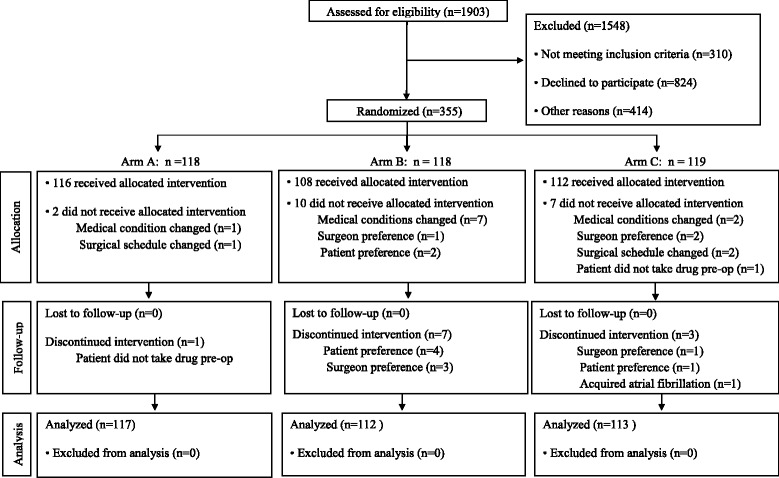
Patient enrollment flowchart

There were 3,860 patients were identified for this study by mailings. There was no response from 1,957 patients. Figure 1 demonstrates the flowchart for the remaining 1,903 patients.

Randomization was balanced between the three study arms for hip vs. knee surgery, and for gender, age, BMI and baseline blood studies. (see Tables 3, 4 and 5) Two patients had rheumatoid arthritis. Both were among the hip replacement patients. One of these had surgery for steroid induced avascular necrosis. All other patients had surgery for osteoarthritis.

**Table 3:** Results of randomization

	Arm A	Arm B	Arm C	Total
	n	n	n	n
Hip	54	64	55	173
Knee	64	54	64	182
Total	118	118	119	355

**Table 4:** Baseline demographics

Demographics		Arm A	Arm B	Arm C	A vs B	A vs C	B vs C
					*p* value	*p* value	*p* value
Hips	% Male & Female	31.4/32.4	36.0/31.0	32.6/36.6	0.646	0.827	0.490
	Age (yr. ± 1 SD)	59.5 (8.6)	60.5 (6.8)	58.9 (9.2)	0.764	0.939	0.553
	BMI (± 1SD)	29 (4.5)	30 (4.6)	29.5 (5.6)	0.507	0.837	0.847
Knees	% Male & Female	27.2/37.5	39.5/28.9	33.3/33.7	0.084	0.397	0.369
	Age (yr. ± 1 SD)	63.5 (8.2)	63.3 (8.2)	62.3 (8.0)	0.985	0.694	0.791
	BMI (± 1 SD)	30.9 (5.9)	31.2 (5.1)	30.8 (5)	0.954	0.993	0.915
Total cohort	% Male & Female	29.3/35.4	37.7/29.7	32.9/34.9	0.111	0.621	0.263
	Age (yr. ± 1 SD)	61.6 (8.6)	62 (7.7)	60.7 (8.7)	0.934	0.687	0.467
	BMI (± 1SD)	30 (5.4)	30.6 (4.9)	30.2 (5.3)	0.618	0.960	0.783

**Table 5:** Baseline laboratory results

Laboratory tests		ARM A	ARM B	ARM C	A vs B	A vs C	B vs C
					*p* value	*p* value	*p* value
Routine laboratory units (± 1 Standard Deviation)							
Hips	Hemoglobin	13.9 (1.2)	14.0 (1.4)	13.7 (1.5)	0.921	0.765	0.525
	Platelets (x 1,000)	245.3 (51.8)	244.2 (53.8)	252.3 (47.0)	0.993	0.753	0.685
	BUN	17.2 (5.0)	17.5 (4.8)	18.0 (7.3)	0.963	0.722	0.865
	Creatinine	0.9 (0.2)	0.9 (0.2)	0.9 (0.2)	0.920	0.692	0.448
	Bilirubin	0.5 (0.2)	0.5 (0.3)	0.5 (0.2)	0.689	0.914	0.434
	ALT	9.3 (8.5)	11.9 (10.8)	11.5 (8.1)	0.294	0.432	0.962
	AST	21.9 (7.0)	22.0 (6.3)	21.9 (5.8)	0.998	0.999	0.994
	PTT	27.8 (2.3)	27.4 (2.4)	27.8 (2.2)	0.636	0.993	0.701
	INR	1.02 (0.05)	1.01 (0.06)	1.02 (0.06)	0.800	0.099	0.814
Knees	Hemoglobin	13.6 (1.5)	13.9 (1.5)	13.6 (1.3)	0.430	0.994	0.493
	Platelets (x 1,000)	257.8 (59.9)	240.0 (47.0)	244.5 (54.6)	0.153	0.346	0.888
	BUN	17.9 (5.0)	17.6 (5.4)	18.6 (5.7)	0.963	0.752	0.589
	Creatinine	0.8 (0.2)	0.9 (0.2)	0.9 (0.2)	0.434	0.143	0.786
	Bilirubin	0.4 (0.3)	0.5 (0.2)	0.5 (0.3)	0.416	0.934	0.638
	ALT	12.6 (12.6)	12.8 (12.0)	12.2 (9.7)	0.995	0.970	0.943
	AST	22.9 (8.7)	22.3 (8.0)	22.4 (6.6)	0.899	0.929	0.997
	PTT	27.6 (2.4)	28.5 (2.3)	27.8 (1.8)	0.067	0.872	0.192
	INR	1.00 (0.05)	1.02 (0.04)	1.02 (0.05)	0.217	0.289	0.985
Total cohort	Hemoglobin	13.7 (1.4)	13.9 (1.4)	13.7 (1.4)	0.464	0.921	0.259
	Platelets (x 1,000)	252.1 (56.5)	242.0 (50.1)	248.1 (51.2)	0.301	0.829	0.640
	BUN	17.6 (5.0)	17.6 (5.1)	18.3 (6.4)	1.000	0.542	0.536
	Creatinine	0.9 (0.2)	0.9 (0.2)	0.9 (0.2)	0.790	0.123	0.392
	Bilirubin	0.5 (0.3)	0.5 (0.2)	0.5 (0.2)	0.292	1.000	0.293
	ALT	11.1 (11.0)	12.4 (11.4)	11.8 (9.0)	0.600	0.853	0.905
	AST	22.4 (8.0)	22.2 (7.3)	22.1 (6.2)	0.946	0.943	1.000
	PTT	27.7 (2.3)	28.0 (2.4)	27.8 (2.0)	0.596	0.957	0.770
	INR	1.01 (0.06)	1.02 (0.05)	1.02 (0.06)	0.767	0.563	0.943

Table 6 demonstrates the patients who withdrew completely from the study and those who withdrew from the study drug but completed the bilateral leg ultrasound as required by the protocol. All patients were included in the Intention to Treat analysis.

**Table 6:** Patient withdrawals from study

	Arm A	Arm B	Arm C
Patients withdrawn from study before starting study drug	1	3	5
Patients withdrawn from study after starting study drug	0	3	1
Patients withdrawn before starting study drug; completed ultrasound	1	7	2
Patients withdrawn after starting study drug; completed ultrasound	1	4	2

### Prothrombin times reported as International Normalized Ratios (INR)

**Fig. 2:**
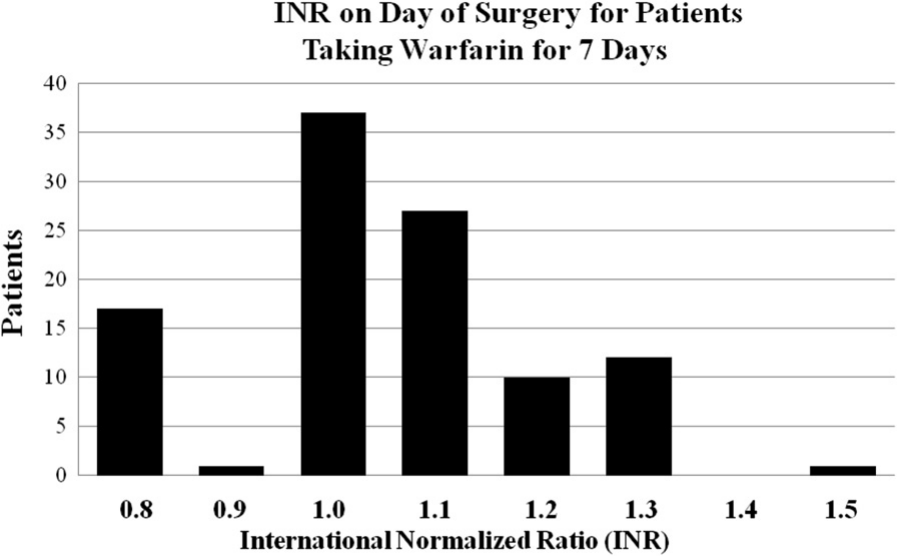
INR’s for patients on Arm C after taking 1 mg warfarin for 7 days before surgery

**Fig. 3:**
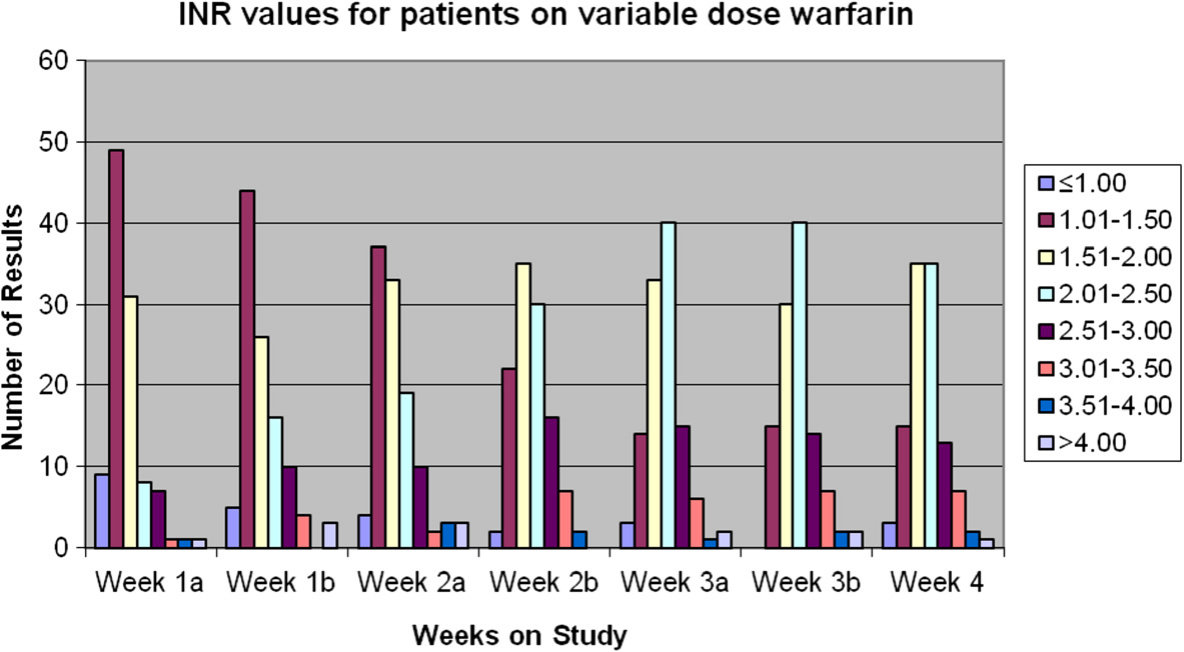
INRs for patients in Arm A receiving variable dose warfarin. These patients were studied twice per week during weeks 1, 2 and 3, as indicated by “week 1a” and “week 1b”, etc

**Fig. 4:**
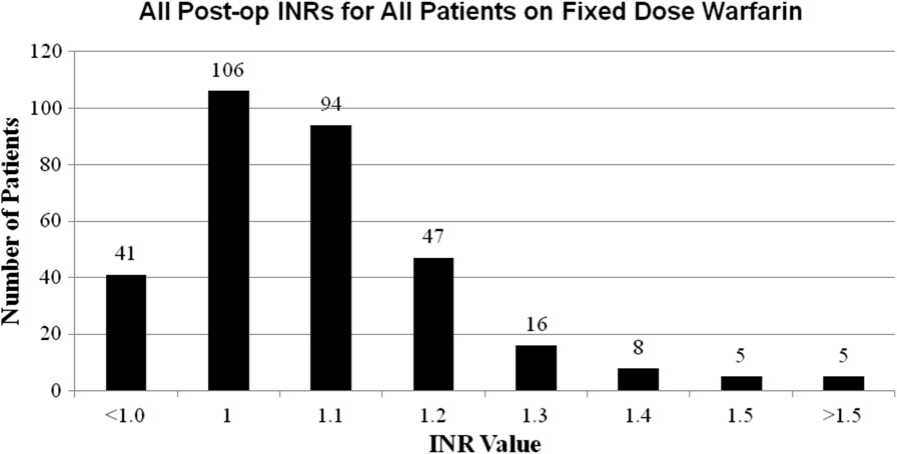
All INRs for patients on Arm C

The INR’s on the day of surgery for patients in Arm C after taking 1.0 mg of warfarin for seven days were ≤ 1.3, with one exception to 1.5. (see Fig. 2) Fig. 3 demonstrates the INR results for patients in Arm A. Each was studied twice per week while on study and the warfarin dose was adjusted according to protocol guidelines. Fig. 4 demonstrates the weekly INR for all patients on Arm C. Five out of 322 INR results that were greater than 1.5.

### Patients receiving HES or NSAIDs

Patients having hip replacement were more likely to be given HES compared to those having knee replacement. (*p* <0.05), but there were no differences among the three study arms. (see Table 7) There were no differences within the study groups relative to use of NSAID’s. (data available).

**Table 7:** Patients receiving hydroxyethyl starch

	Arm A	Arm B	Arm C	A vs. B	A vs C	B vs C
	n out of total	n out of total	n out of total	*p*-value	*p*-value	*p*-value
Hips	21 out of 53 (39.6 %)	18 out of 54 (33.3 %)	17 out of 52 (30.4 %)	0.500	0.461	0.944
Knees	1 out of 62 (12.5 %)	2 out of 63 (25 %)	5 out of 62 (62.5 %)	0.576	0.131	0.251
Total	22 out of 115 (19.1 %)	20 out of 117 (17.1 %)	22 out of 114 (19.3 %)	0.687	0.974	0.664

### Protocol violations

There were 73 minor protocol violations and 24 major protocol violations, all equally distributed among the study arms (data available) No study violation was associated with an adverse event.

### Composite endpoints

Complete bilateral leg ultrasounds were performed for 342 patients distributed equally in the study arms. (data available) There were no patients with VTE at time of discharge. Two patients in Arm C had delayed, asymptomatic, distal ultrasound-detected deep vein thrombosis. One of these patients had knee replacement surgery and the second had hip replacement surgery. They were discharged on days 4 and 5 post-operative respectively. There were no pulmonary emboli and no deaths. The *p*-values, the Exact Odds ratios with associated confidence limits, the non-inferiority analysis and the 95 % confidence limits are shown in Table 8.

**Table 8:** Composite endpoints

	Arm A	Arm B	Arm C
	n	n	n
DVT proximal	0	0	0
DVT distal	0	0	2
PE	0	0	0
Death on study	0	0	0
Exact Odds Ratios			
Unadjusted	Estimate	95 % Confidence Limits	*p*-value
Odds Ratio	(median unbiased)		
Arm A vs. Arm C	0.397	0 – 3.344	0.480
Arm B vs. Arm C	0.387	0 - 3.259	0.476
Noninferiority Analysis of Risk Difference			
Risk Difference	Estimate	95 % Confidence Limits	*p*-value*
Arm C vs. Arm A	−0.0180	−0.0428 – 0.0067	<0.0001
Arm C vs. Arm B	−0.0180	−0.0428 – 0.0067	<0.0001

*Using a noninferiority limit of -0.015

Arm C was not to be different from Arms A and B. Because Arm C was non-inferior to Arms A or B, we analyzed equivalency between Arm C and the two arms using the Wald Method for the non-inferiority. Analysis of risk differences between the Arm C minus Arm A and Arm B showed that Arm C is also equivalent to both arm A and arm B, at an equivalency margin of 15 %. (*p*-value <0.0001; one-sided 95 % CI: -0.0428–0.0067) Thus, there are no statistical differences between Arm C and the other arms of the study.

### Adverse events

There were no differences for the surgical estimated blood loss (EBL) or transfusions given among the three study groups. (data available) Patients taking NSAIDs did not have increased hemorrhagic complications. (data available) There were no surgery-related complications such as fracture, dislocation or infection.

More patients in Arm A reported a hemorrhagic event as compared to Arms B and C. The risk of reporting any hemorrhagic complication in Arm A was 1.29 times that of Arm B (95 % CI: 0.61–2.71), and was 1.54 times that of those in Arm C (95 % CI: 0.71–3.33). While there were differences for patients having knee replacement surgery these comparisons did not reach statistical significance for the study groups. (see Table 9) There were no differences of hemorrhagic complications using unadjusted and adjusted odds ratio related to study arm or related to hip vs. knee surgery, adjusted for effects of age, gender and use of HES and NSAID’s. (see Tables 10 and 11) One Arm C patient developed ischemic colitis complicated by gross hematochezia. Despite his taking only 1 mg warfarin daily the INR was prolonged. He was then found to have homozygous vitamin K epoxide reductase–1 (VKORC-1) genome disorder. The event was managed and he remained on study.

**Table 9:** All hemorrhagic complications including all minor and major bleeds

	Arm A	Arm B	Arm C	A vs. B	A vs C	B vs C
	n out of total	n out of total	n out of total	*p*-value	*p*-value	*p*-value
Hips	9 out of 54	15 out of 54	6 out of 55	0.169	0.386	0.030
	(16.7 %)	(27.8 %)	(10.9 %)			
Knees	20 out of 64	8 out of 64	12 out of 64	0.013	0.105	0.333
	(31.2 %)	(12.5 %)	(18.7 %)			
Total	28 out of 118	23 out of 118	18 out of 119	0.347	0.070	0.375
	(23.7 %)	(19.5 %)	(15.1 %)

**Table 10:** Unadjusted estimated effect of treatment on hemorrhagic complications

		Unadjusted OR (95 % CI)	*p*-value
Fondaparinux vs. variable dose warfarin	Both	1.29 (0.61-2.71)	0.709
	Hip	0.45 (0.14-1.42)	0.236
	Knee	3.18 (1.07-9.45)	0.034
Fixed dose warfarin vs. variable dose warfarin	Both	0.83 (0.38-1.84)	0.854
	Hip	0.38 (0.12-1.24)	0.134
	Knee	1.78 (0.57-5.62)	0.463
Fixed dose warfarin vs. fondaparinux	Both	0.65 (0.30-1.40)	0.384
	Hip	0.84 (0.23-3.10)	0.946
	Knee	0.56 (0.21-1.47)	0.338

**Table 11:** Estimate of treatment effect of all hemorrhagic complication stratified for hip vs knee adjusted for effects of age, gender, and NSAID and hetastarch used

		Adjusted odds ratio(95 % CI)	*p*-value
Hip	Fondaparinux vs. variable dose warfarin	0.45 (0.14-1.44)	0.238
	Fixed dose warfarin vs. variable dose warfarin	0.40 (0.12-1.32)	0.170
	Fixed dose warfarin vs. fondaparinux	0.89 (0.24-3.3645)	0.978
Knee	Fondaparinux vs. variable dose warfarin	3.45 (1.15-10.32)	0.022
	Fixed dose warfarin vs. variable dose warfarin	1.78 (0.56-5.68)	0.471
	Fixed dose warfarin vs. fondaparinux	0.52 (0.20-1.36)	0.248

Four major adverse events occurred. Table 12 demonstrates the distribution of these events as being possibly related, probably related or related to study drugs. The two “probably related” leg hematomas occurred while the patients were in the hospital. The other two events occurred following discharge from the hospital.

**Table 12:** Major complications related to study drugs

Relationship to study drug	n	Description	Study arm
Possibly related	1	Painless hematochezia following hard bowel movement (minor)	B
Probably related	1	Hematoma, operative leg, from post-op trauma (minor; delayed discharge)	B
	1	Hematoma evaluated, no intervention required (minor; delayed discharge)	B
Related	1	Hemorrhage with ischemic colitis. INR prolonged on 1 mg warfarin	C
		per day due to VKORC-1 genome disorder. (major; readmitted to hospital)	

### D-dimer

Patients having knee replacement were more likely to have elevated D-dimer at the completion of the study compared to those having hip surgery. (*p* <0.0001) (see Table 13) There were no differences relative to the arms of the study. There were no delayed onset VTE events after 6 and 12 months from completion of study.

**Table 13:** Changes of D-dimer from baseline negative/indeterminate to >1000 μgm/dl after 28 ± 2 days

	Changed from 0 – 999 at baseline to >1,000 after 28 ± 2 days					
	Yes	No	*p*-value for hip vs. knee			
	n (%)	n (%)				
Hips	45 (31.5 %)	98 (68.5 %)	<0.0001			
Knees	116 (70.7 %)	48 (29.3 %)				
Patients with elevated D-dimer (>1,000 μg/dL) at follow-up, stratified for knee vs. hip and study arm						
	Arm A	Arm B	Arm C	A vs. B	A vs. C	B vs. C
				*p*-value	*p*-value	*p*-value
Knees	12 of 47 (25.5 %)	15 of 50 (30.0 %)	18 of 46 (39.1 %)	0.624	0.163	0.348
Hips	41 of 54 (75.9 %)	38 of 59 (64.4 %)	37 of 51 (72.5 %)	0.185	0.693	0.362
Total	53 of 101 (52.5 %)	53 of 109 (48.6 %)	55 of 97 (56.7 %)	0.577	0.551	0.247

## Discussion

Patients given no anticoagulants after hip or knee arthroplasty experience high rates of postoperative VTE [23–28]. Heretofore, commonly used anticoagulants reduced the frequency of postoperative VTE to 4–6 %. A meta-analysis of modern low molecular weight heparins, oral Xa inhibitors or oral IIa inhibitors included 47 clinical trials and observational studies involving 44,844 patients demonstrated rates of symptomatic DVT prior to discharge of 1.09 % for patients having TKA and 0.53 % for patients having THA, with heterogeneity of results among the included reports [29]. The current study shows that our fixed low dose warfarin regimen approximates the same level of benefit.

The two agents used in this current study as controls can be considered as among the available standards-of-care agents available for DVT prophylaxis. Variable dose warfarin has been used for many years, and is effective with the current target range of INR [30, 31]. Fondaparinux, a synthesized pentasaccaride with anti-factor Xa activity, was among the newer agents with VTE prophylaxis activity when this study was designed [22, 32–35].

As in our previous orthopedic studies and as with other investigators we selected a preoperative lead-time of 7 days for the fixed low dose warfarin used in this study [8, 11, 36, 37]. In an earlier central venous catheter study we choose 3 days [6]. We would not expect this low dose warfarin therapy to succeed if started following surgery, even though in one series it did succeed even when therapy was begun after central venous catheter insertion [12, 38–41].

There were two previous smaller but similarly structured studies that derived negative results for low dose warfarin following major orthopedic surgery. Poller, et al., in a report of 68 evaluable patients randomized to receive either warfarin 1 mg daily starting 7 days preoperatively or 5,000 units heparin starting 2 h preoperatively, with venography as the diagnostic tool [36]. Fordyce, et al., studied 148 patients having primary hip replacement surgery were randomized to take 1 mg warfarin starting 7 days prior to surgery or to be non-treated controls. Iodine-125 labeled fibrinogen uptake (with venography confirmation for positive femoral vein uptake) was used for detection of DVT [37].

Differences in outcomes may be dependent upon the DVT detection techniques applied. Color Doppler ultrasound was used in this study to detect DVT. Ultrasound imaging is accepted for diagnosing DVT with sensitivity and specificity for detection of proximal deep vein thrombosis is over 95 %. In studies of asymptomatic patients, the sensitivity the test has been variable. Nevertheless, a single negative complete lower limb examination is considered sufficient to exclude clinically important DVT [42–44]. New symptoms always require further imaging. In the current study patients were called at 6 and 12 months to detect any delayed symptomatic VTE events in order to detect any false negative results.

On the other hand, venography, often considered the gold standard for DVT detection studies, is far from the ideal diagnostic procedure. Venography-dependent studies often have significant numbers of patients who never get the venogram, have unilateral studies instead of bilateral studies, or are rejected by the adjudicating committees as technically inadequate [24, 45–50]. Venography also involves added risks for patients with allergic and renal reactions, skin necrosis if contrast dye extravasates and post-injection phlebitis in up to 2 % of patients in whom conventional ionic contrast agents are used. Even with use of lower osmolar contrast agents, minor side effects occur in about 20 % of patients undergoing venography. Fibrinogen scanning may have been supersensitive.

Also, the current study was performed in a hospital with a high volume of joint replacement surgery, wherein optimal non-pharmacologic DVT prophylaxis is also applied along with chemoprophylaxis. Patients are told to discontinue smoking and estrogen supplements prior to surgery, to walk the day of surgery, and wear bilateral pneumatic compression stockings in hospital followed by elastic compression stockings after discharge until follow-up visits. Perhaps some of these elements may contribute to differences between the study outcomes.

There are clinically relevant elements within any study that cannot be stratified or controlled. In this study tourniquets were used for all patients having knee replacement, and it is assumed that the time the tourniquets were applied was equal for each study group [50]. Volume expansion by crystalloids can influence post-operative coagulation; it is assumed that the volume expansions were equivalent for each study group [51]. HES can have effects upon the clotting system [52, 53]. There were no differences for the use of HES among the three arms of this study. Also, as there are over 100 drugs, foods and supplements reported to interfere with platelet function, with unclear clinical significance for most, it would have been difficult to control for all of these agents [54]. It was decided by the protocol writing committee to track and analyze six of the more commonly used NSAIDs.

A positive D-dimer assay after conclusion of therapy for acute DVT or PE has been previously reported to be predictive of relapse of VTE. In one such study following one month of therapy for DVT, 39 % of patients had elevated D-dimer and seven of these patients went on to have recurrent VTE [55]. There is also increasing recognition that longer prophylactic therapy is beneficial in the post-operative setting [56–62]. The current study prescribed that all study patients have measurements of D-dimer at the end of the treatment. No patient had delayed onset VTE when called 6 months and 12 months following completion of one month of therapy. Thus, in this study a positive D-dimer at the conclusion of therapy had no predictive value for delayed onset VTE after prolonger therapy of one month.

This proof of concept study builds upon prior studies examining low dose schedules for postoperative VTE chemoprophylaxis with warfarin. Trail limitations include that this is a single institution, open label but assessor-blind study study. A similar multi-institutional study with a larger number of patients would be welcomed. Perhaps it would also be useful to attempt the next such study with combined Doppler ultrasound and venography detection for DVT to confirm adequacy of Doppler ultrasound.

In conclusion, the three regimens in this study offer effective prophylaxis against thromboembolic disease following elective arthroplasty of hips and knees. It appears that fixed low dose warfarin when used as described is equivalent to two of the more intense regimens. By extending postoperative anticoagulation to four weeks there appears to be less chance for delayed VTE, even if there is a positive D-dimer at conclusion of treatment. Each regimen carries some risk and some inconvenience for the patient. The fixed low dose warfarin regimen herein described saves the patients the inconvenience of multiple blood draws, but does not eliminate that need completely, as demonstrated in our patient with vitamin K epoxide reductase genome disorder. Nevertheless, it should carry minimal hemorrhagic potential and require reduced laboratory support. It should be less expensive and easier to administer than other protocols using anti-Xa and anti-IIa agents, which has implications for health care costs.
